# Integrating Blood-Based Immune-Inflammation Biomarkers into Artificial Intelligence–Driven Prognostic Models in Oncology

**DOI:** 10.3390/ijms27073192

**Published:** 2026-03-31

**Authors:** Agata Dobrowolska-Szumowska, Zbigniew Krzysztof Kamocki, Żaneta Anna Mierzejewska

**Affiliations:** 1 Second Department of General and Gastroenterological Surgery, Medical University of Bialystok, M. Sklodowskiej-Curie 24A, 15-276 Bialystok, Poland; agata.dobrowolska@umb.edu.pl (A.D.-S.); zbigniew.kamocki@umb.edu.pl (Z.K.K.); 2 Institute of Biomedical Engineering, Faculty of Mechanical Department, Bialystok University of Technology, Wiejska 45C, 15-351 Bialystok, Poland

**Keywords:** systemic immune-inflammation index (SII), survival analysis, artificial intelligence, explainable AI, prognostic biomarkers, host-related factors, precision oncology, immune-inflammatory markers, risk stratification, translational oncology

## Abstract

Stratification in oncology remains largely based on tumor-intrinsic characteristics, with limited integration of systemic host biology. The Systemic Immune-Inflammation Index (SII), derived from peripheral blood counts, reflects interactions between inflammation, thrombosis, and immune competence and has emerged as a simple, accessible prognostic biomarker. At the same time, artificial intelligence (AI)–based survival models enable integration of heterogeneous data and nonlinear relationships in outcome prediction. However, the structured incorporation of biologically interpretable host-derived markers into explainable AI frameworks remains insufficiently defined. A structured narrative review of PubMed/MEDLINE and Scopus was conducted to examine (i) the prognostic role of SII in solid tumors and (ii) AI-based survival modeling in oncology. Rather than quantitative synthesis, the review prioritized methodological quality, biological plausibility, validation strategies, and explainability relevant to clinical translation. Elevated SII is consistently associated with poorer survival across multiple solid malignancies and treatment contexts, although effect sizes vary and methodological heterogeneity remains considerable. AI-based survival models can incorporate continuous and multimodal variables, capture nonlinear associations, and model complex tumor–host interactions. We propose a translational framework integrating SII into explainable AI survival pipelines through continuous modeling, interaction-aware feature engineering, rigorous internal and external validation, and interpretable modeling approaches. Embedding SII as a biologically grounded continuous variable within multivariable prognostic architectures may enhance personalized risk stratification in precision oncology, pending prospective validation and standardized reporting.

## 1. Introduction

Accurate prognostic stratification remains a central challenge in contemporary oncology and a prerequisite for personalized treatment planning. Despite advances in molecular profiling, targeted therapies, and immunotherapy, clinical decision-making continues to rely largely on conventional systems such as tumor–node–metastasis (TNM) staging, histopathological grading, and selected molecular alterations. Although these parameters characterize tumor burden and biological subtype, they only partially explain interpatient heterogeneity [1,2,3].

Cancer progression and survival are influenced not only by tumor-intrinsic features but also by host-related biological processes, particularly systemic immune and inflammatory responses. Chronic inflammation promotes tumor progression through mechanisms such as genomic instability, angiogenesis, and immune evasion [4], whereas effective lymphocyte-mediated immunity is associated with improved outcomes across malignancies [5]. These observations have increased interest in biomarkers reflecting systemic immune status.

Peripheral blood–derived markers provide a practical approach to assessing systemic immune response. Ratios such as the neutrophil-to-lymphocyte ratio (NLR), platelet-to-lymphocyte ratio (PLR), and lymphocyte-to-monocyte ratio (LMR) have demonstrated prognostic associations across tumor types, but each captures only a limited aspect of the inflammatory response [6,7,8,9].

The Systemic Immune-Inflammation Index (SII), calculated from neutrophil, platelet, and lymphocyte counts, integrates inflammation, thrombosis, and immune competence into a single measure [7,10]. Elevated SII has been consistently associated with poorer survival across multiple solid tumors, although effect sizes vary and methodological heterogeneity remains substantial [11,12,13]. Parallel, artificial intelligence (AI)–based survival models enable integration of heterogeneous clinical, laboratory, and molecular data and capture nonlinear relationships. However, their performance is context-dependent, and improvements over conventional models are not consistent across studies [14,15,16,17].

Despite growing interest in both immune-inflammatory biomarkers and AI-based modeling, these domains are often studied separately [11,14,17,18]. Structured approaches for incorporating biologically interpretable host-derived markers into explainable and clinically validated AI models remain limited, particularly in light of concerns regarding methodological heterogeneity and risk of bias in prognostic modeling research [17,19,20].

This review adopts an integrative perspective and proposes a structured framework for incorporating SII into multivariable, explainable AI-based survival models. Rather than focusing on algorithm comparison, we emphasize biological rationale, methodological principles, and validation strategies relevant to clinical translation. This approach aims to align systemic immune biology with contemporary standards of prognostic modeling and improve the interpretability and applicability of AI-driven risk stratification in oncology.

The search strategy combined controlled vocabulary and free-text terms related to (i) “systemic immune-inflammation index” OR “SII” OR “immune-inflammatory biomarkers” AND (ii) “artificial intelligence” OR “machine learning” OR “deep learning” OR “survival analysis” OR “time-to-event modeling” AND (iii) “cancer” OR “oncology”. Searches were conducted in PubMed/MEDLINE and Scopus for studies published between January 2010 and January 2026.

Retrieved records were screened for relevance to oncologic survival outcomes and the methodological integration of immune-inflammatory biomarkers or AI-based modeling approaches. Priority was given to studies providing methodological detail, multivariable analyses, or validation procedures. The final body of literature included studies addressing the prognostic role of SII across multiple solid malignancies as well as methodological research on AI-based survival modeling.

The included SII literature covered hepatocellular carcinoma, pancreatic cancer, colorectal cancer, gastric cancer, lung cancer, urothelial carcinoma, breast cancer, and other solid tumors, with the largest representation observed in gastrointestinal and thoracic oncology cohorts.

Given the narrative and framework-oriented objective of this review, formal quantitative synthesis of effect estimates was not performed. Risk of bias was not assessed using structured instruments such as PROBAST, as the aim was conceptual integration rather than systematic evidence grading. Consequently, the findings should be interpreted as a structured conceptual synthesis rather than a formal systematic evaluation of evidence.

## 2. Prognostic Biomarkers in Oncology: From Tumor-Centered to Host-Related Factors

### 2.1. Tumor-Centered Prognostic Paradigms and Their Limitations

Prognostic assessment in oncology has traditionally relied on tumor-focused parameters, including tumor size, nodal status, metastatic spread, histopathological subtype, grading, and selected molecular alterations. These variables underpin staging systems and therapeutic algorithms across malignancies and remain essential for estimating tumor burden and intrinsic aggressiveness [21,22].

However, tumor-centered approaches incompletely account for the heterogeneity observed among patients with similar clinicopathological profiles. Individuals with comparable stage and molecular characteristics may exhibit markedly different survival outcomes and treatment responses [23]. This suggests that tumor-intrinsic features alone do not fully determine disease course.

In addition, conventional staging systems are largely static and may not adequately reflect biological processes that evolve during disease progression or under therapeutic pressure [24]. For example, they do not capture temporal changes in systemic immune response, treatment-induced alterations in tumor biology, or dynamic tumor–host interactions that may influence clinical outcomes. As oncology advances toward adaptive and individualized care, prognostic strategies increasingly require integration of tumor characteristics with systemic biological context.

### 2.2. Host-Related Immune-Inflammatory Biomarkers

Recognition of the host immune system as an active determinant of cancer progression has expanded interest in systemic immune-inflammatory biomarkers. Chronic inflammation contributes to multiple hallmarks of cancer, including genomic instability, angiogenesis, immune evasion, and metastatic dissemination [25,26]. Conversely, effective antitumor immune surveillance, particularly lymphocyte-mediated adaptive responses, is associated with improved outcomes across malignancies [5].

Peripheral blood–derived markers provide a practical means of assessing systemic immune status. Complete blood counts are routinely available and reflect circulating inflammatory and immune cell populations [27]. Ratios such as the neutrophil-to-lymphocyte ratio, platelet-to-lymphocyte ratio, and lymphocyte-to-monocyte ratio have shown prognostic associations in multiple tumor types and treatment settings [28,29,30].

These indices, however, capture only pairwise cellular relationships and may not represent the broader immune-inflammatory milieu [7,30]. Their performance varies across disease stages and therapeutic contexts, highlighting the need for more integrative measures.

Composite indices incorporating multiple hematologic compartments have, therefore, been proposed to provide a more comprehensive representation of systemic immune-inflammatory status. Among these, the Systemic Immune-Inflammation Index (SII) has been extensively studied and demonstrates consistent prognostic associations across solid malignancies [31,32,33]. For clarity, the most commonly used peripheral blood–based immune-inflammatory biomarkers and their key biological interpretations are summarized in Table 1.

### 2.3. Emerging Prognostic Biomarkers and Multimodal Risk Stratification

Recent advances in oncology have expanded the prognostic biomarker landscape beyond conventional clinicopathological and hematological parameters toward molecular and computational approaches. Circulating tumor DNA enables detection of minimal residual disease, early relapse prediction, and longitudinal monitoring of treatment response [34]. Similarly, immune profiling, including tumor-infiltrating lymphocytes, peripheral immune cell subsets, and cytokine signatures, provides insight into antitumor immunity and therapeutic susceptibility, particularly in the context of immunotherapy [35].

Radiomics and radiogenomics extract quantitative features from routine imaging and link them to tumor heterogeneity and clinical outcomes. Multi-omics approaches integrating genomic, transcriptomic, proteomic, and metabolomic data further enhance characterization of disease biology [36,37,38].

Despite their depth, many of these approaches are limited by cost, technical complexity, and restricted availability in routine practice [39,40]. In addition, they primarily capture tumor-intrinsic properties and may only indirectly reflect systemic immune status. In this context, peripheral blood–based indices such as the Systemic Immune-Inflammation Index (SII) provide accessible information on host-related biological processes and may complement tumor-derived data [11,41,42].

From a modeling perspective, AI-based prognostic systems must be aligned with both the intended clinical application and the characteristics of available data [43,44]. Variables that are widely available, reproducible, and biologically interpretable are particularly valuable in this context [17]. Immune-inflammatory indices such as SII meet these criteria and may function as stabilizing features within multivariable models. Unlike high-dimensional molecular data, these biomarkers are routinely measured, relatively standardized, and less sensitive to technical variability. They, therefore, provide consistent signals that can enhance model robustness and generalizability across datasets. Their interpretability also aligns with the increasing emphasis on explainable artificial intelligence, facilitating clinical trust and potential implementation [11].

## 3. The Systemic Immune-Inflammation Index (SII)

### 3.1. Definition and Conceptual Framework

The Systemic Immune-Inflammation Index (SII) is a composite biomarker derived from routinely available peripheral blood parameters [45] and is calculated as follows:

SII = (Platelet count × Neutrophil count)/Lymphocyte count

SII integrates three key hematologic compartments involved in cancer-related processes: neutrophils (inflammation), platelets (thrombosis and tumor promotion), and lymphocytes (adaptive immunity) [46]. It reflects the balance between pro-inflammatory and immunosuppressive mechanisms and antitumor immune surveillance [7,11].

Importantly, SII does not provide pathway-specific mechanistic information. Rather, it serves as a summary indicator of systemic immune-inflammatory status associated with cancer progression and survival [47]. It should, therefore, be interpreted within a multivariable clinical context rather than as a mechanistically specific marker.

Compared with other systemic inflammatory markers, such as C-reactive protein (CRP), the modified Glasgow Prognostic Score (mGPS), or the Prognostic Nutritional Index (PNI), SII integrates multiple hematologic components into a single measure [48,49]. However, as an indirect surrogate of inflammation, it may capture overlapping biological processes, and comparative evidence remains limited [50].

### 3.2. Biological Rationale: Linking Inflammation, Thrombosis, and Immune Suppression

The biological plausibility of SII as a prognostic biomarker is supported by extensive evidence implicating neutrophils, platelets, and lymphocytes in cancer-related processes. These cell populations contribute to inflammation, tumor progression, and immune regulation. Neutrophils are key mediators of systemic inflammation and promote tumor progression through the release of cytokines, proteases, and reactive oxygen species [51]. They also form neutrophil extracellular traps, which facilitate tumor cell adhesion, immune evasion, and metastatic dissemination [52].

Platelets contribute to tumor progression by protecting circulating tumor cells from immune clearance, enhancing endothelial adhesion, and releasing pro-angiogenic factors [53,54,55]. Increased platelet activity has been associated with adverse outcomes across multiple malignancies.

**Figure 1:**
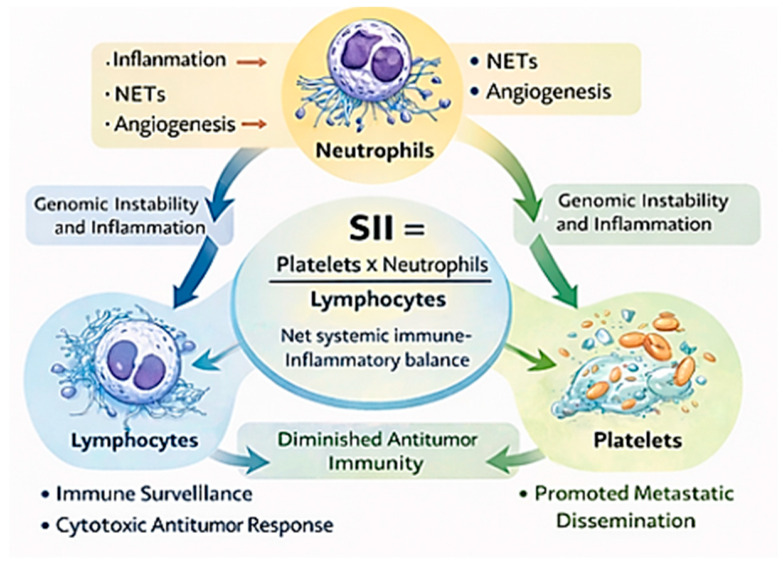
Biological rationale of the Systemic Immune-Inflammation Index. This figure was created in BioRender. Mierzejewska, A. (2026) (Available online: https://BioRender.com/3pzt40f; accessed date: 13 March 2026).

In contrast, lymphocytes, particularly cytotoxic T cells, mediate antitumor immunity. Reduced lymphocyte counts may reflect impaired immune surveillance and are associated with poorer clinical outcomes. Elevated SII values, reflecting higher neutrophil and platelet counts relative to lymphocytes, therefore, correspond to a systemic state characterized by inflammation and immune suppression, which is associated with adverse prognosis [31,56,57] (Figure 1).

### 3.3. Clinical and Prognostic Evidence Across Cancer Types and Treatment Contexts

The prognostic relevance of SII has been evaluated across multiple solid malignancies and treatment settings [11,31]. Elevated pre-treatment SII is consistently associated with shorter overall survival and progression-free survival in hepatocellular, pancreatic, colorectal, gastric, and lung cancers [45,47,58,59,60,61].

Most studies report independent associations after multivariable adjustment. However, formal evaluation of incremental predictive value remains limited. In particular, direct comparisons between base clinicopathological models and models extended with SII, using discrimination, calibration, or decision-analytic metrics, are rarely performed. As a result, statistical significance does not necessarily translate into improved risk prediction. As summarized in Table 2, meta-analyses report pooled hazard ratios typically ranging from approximately 1.5 to 2.5, although moderate to substantial heterogeneity is common (I^2^ often > 50%). This variability likely reflects differences in study design, patient populations, disease stage distribution, treatment modalities, and analytical approaches.

Most available studies are retrospective and frequently single-center, which may introduce selection bias and limit external validity [17]. In addition, effect sizes appear to vary by disease stage and availability of molecular stratification markers, suggesting that the incremental contribution of SII is context-dependent.

Across treatment settings, including surgery, chemotherapy, radiotherapy, targeted therapy, and immunotherapy, elevated SII has generally been associated with poorer outcomes [62,63,64]. Although several studies have explored its potential relevance in patients receiving immune checkpoint inhibitors [58,65,66], formal interaction analyses demonstrating treatment-specific effects remain limited. Current evidence, therefore, supports a primarily prognostic rather than predictive role [67,68].

From a methodological perspective, it is important to distinguish between prognostic and predictive biomarkers. Prognostic markers provide information on outcome independent of treatment, whereas predictive biomarkers identify differential treatment benefit. Without formal interaction testing, interpretation of SII as a predictive marker remains premature.

Overall, SII demonstrates consistent prognostic associations across cancer types. However, robust evidence of incremental predictive value beyond established models remains limited, and further validation in well-designed prospective studies is required.

### 3.4. Methodological, Analytical, and Biological Limitations

Despite consistent associations with clinical outcomes, several factors limit the clinical application of SII. A major challenge is the lack of standardized cut-off values for risk stratification [69]. Reported thresholds vary across studies due to differences in patient populations, laboratory practices, and statistical methods, which complicates comparison and clinical implementation [32,33].

Most available evidence is derived from retrospective cohorts, introducing potential selection bias and confounding [11,17]. In addition, SII is influenced by non-malignant conditions such as infection, chronic inflammatory disorders, and concomitant medications, limiting its specificity for cancer-related processes. Single baseline measurements may also fail to reflect temporal changes during treatment or disease progression [46,69].

From a statistical perspective, dichotomization remains common but results in information loss and may inflate effect estimates [70,71]. Data-driven threshold selection can introduce optimism bias and reduce transportability. Modeling SII as a continuous variable may mitigate these limitations.

Meta-analyses frequently report moderate to high heterogeneity and potential publication bias [72,73,74]. External validation remains limited, further constraining generalizability. Beyond baseline assessment, longitudinal evaluation of SII using serial measurements may provide additional insight into dynamic tumor–host interactions. Time-dependent modeling approaches could capture treatment-related changes and evolving systemic immune status, potentially improving risk stratification over the disease course. However, the clinical utility of such approaches requires prospective validation.

Overall, SII should not be used as an isolated risk classifier. Its clinical value is better assessed within multivariable, externally validated prediction models that account for continuous effects and context-dependent variability [75,76,77,78].

## 4. Artificial Intelligence in Prognostic Modeling of Cancer Outcomes

### 4.1. From Traditional Statistical Models to Data-Driven Prognostication

Prognostic modeling in oncology has traditionally relied on statistical approaches, most notably the Cox proportional hazards model. These models are widely used due to their interpretability and well-established theoretical foundation. However, they rely on assumptions such as proportional hazards and predefined relationships between variables, which may not fully capture the complexity of cancer progression [79].

Cancer outcomes reflect interactions among tumor characteristics, host-related factors, treatment variables, and temporal dynamics [80,81,82]. Modeling these relationships within predefined regression structures may require simplification and can limit the representation of nonlinear or higher-order effects [83].

Machine learning and deep learning approaches provide greater flexibility by accommodating nonlinear relationships and interactions without explicit prespecification [81,84]. These methods can integrate heterogeneous data sources and identify patterns that may not be captured by conventional models [85,86].

However, empirical evidence indicates that machine learning does not consistently outperform well-specified regression models in structured clinical datasets [17,82]. In many cases, Cox-based models achieve comparable discrimination. Therefore, the choice of method should be guided by data characteristics, predictor complexity, and clinical context rather than assumptions of superiority.

Artificial intelligence should be viewed as an extension of traditional modeling rather than a replacement. Its value depends on appropriate application, validation, and interpretability within clinically relevant settings.

### 4.2. Machine Learning Approaches for Time-to-Event Outcomes

Most clinically relevant prognostic questions in oncology involve time-to-event outcomes, such as overall survival, progression-free survival, recurrence, or treatment failure. This has led to the development of machine learning methods adapted to censored survival data [87,88].

Common approaches include random survival forests, gradient boosting methods, and neural network–based survival models [89]. Random survival forests extend tree-based models to survival analysis without relying on proportional hazards assumptions [90]. Boosting methods iteratively optimize predictive performance and can model complex relationships in structured data [91,92,93]. Neural network–based models, such as DeepSurv and DeepHit, allow flexible estimation of risk functions and time-dependent effects [94,95].

These approaches can incorporate continuous predictors and capture nonlinear associations without explicit model specification, which may be relevant for biomarkers such as SII [91,96]. However, increased model flexibility is associated with a higher risk of overfitting, particularly in datasets with limited sample size or event numbers. Robust internal validation and independent external validation are, therefore, essential to ensure generalizability [17].

The choice of modeling approach should be guided by dataset characteristics, predictor dimensionality, and clinical objectives. In structured clinical datasets, simpler and well-calibrated models may perform comparably to more complex methods.

### 4.3. Multimodal Data Integration and Prognostic Performance

A key advantage of AI-based prognostic systems is the ability to integrate multimodal data. In oncology, patient outcomes are influenced by tumor characteristics, systemic factors, treatment variables, and temporal dynamics. Combining these data sources within a single model may improve representation of disease complexity [97,98,99,100].

AI-based approaches can incorporate clinical variables, laboratory parameters, imaging features, and molecular data within a unified analytical framework. This may enable identification of patterns that are not captured by predefined regression structures.

However, reported improvements in predictive performance are generally modest and vary across studies, as illustrated in Table 3 [17,82]. In structured clinical datasets with limited predictor dimensionality, well-specified Cox models often achieve comparable discrimination [68,71,101]. Therefore, the value of multimodal integration depends on data quality, sample size, and clinical context rather than model complexity alone.

Model evaluation should extend beyond discrimination metrics. The concordance index reflects ranking ability but does not assess agreement between predicted and observed outcomes. Calibration is essential for individual risk estimation, yet it remains underreported in many studies [101]. In addition, improvements in statistical performance do not necessarily translate into clinical benefit [102]. Decision curve analysis and related approaches can help determine whether differences in model performance lead to meaningful changes in clinical decision-making.

Incorporating host-related biomarkers alongside tumor-centered variables may improve predictive stability in selected contexts [102]. However, the contribution of individual variables should be evaluated within fully specified multivariable models and validated in independent datasets.

**Table 3:** Comparative performance of Cox and AI-based survival models in oncology.

Author	Cancer Type	N	Model Comparison	C-Index (Cox)	C-Index (AI)	External Validation
Katzman et al. [103]	Breast cancer (METABRIC)	~1980	Cox PH vs. DeepSurv	0.63	0.65	Internal validation
Li et al. [104]	Colorectal cancer	416	Cox variants vs. DeepSurv	0.69	0.77	Internal test set
Li et al. [105]	Early-stage young breast cancer	7850	Cox PH vs. Random Survival Forest	0.70	0.70	Yes (external cohort)
Ma et al. [106]	Pan-cancer (TCGA, 20 types)	Multiple cohorts	Lasso-Cox vs. XGBoost	~0.64–0.66 *	0.69	Cross-validation across datasets
Huang et al. [107]	Ampullary adenocarcinoma	2935	Cox PH vs. DeepSurv	0.69	0.73	Internal test split

* Range across datasets or approximated from reported results.

### 4.4. Interpretability, Explainability, and Clinical Trust

In light of the cited studies, interpretability remains a central barrier to the clinical implementation of machine learning models in oncology. Evidence from studies such as Chalkidis et al. (2023) indicates that even well-performing and externally validated prediction models may have limited clinical utility if their decision-making processes are not transparent to clinicians [108]. Similarly, Collins et al. (2025) emphasize that “black-box” models can undermine clinical trust and hinder adoption, particularly in high-stakes decision-making contexts [109]. In this regard, explainable artificial intelligence (XAI) methods are increasingly recognized as essential for bridging the gap between predictive performance and clinical usability. As highlighted in the scoping review by Santos and Amorim-Lopes (2025), techniques such as feature importance measures and SHAP values can enhance transparency by clarifying how individual variables contribute to model outputs [110]. Moreover, Biziaev et al. (2023) demonstrate that incorporating clinically interpretable predictors within modeling frameworks can further support their practical applicability [111].

However, it is important to note that improved interpretability does not equate to causal understanding. XAI methods describe patterns and associations within the data rather than underlying biological mechanisms. Therefore, as consistently emphasized across these studies, model interpretation must be contextualized within clinical expertise and supported by rigorous external validation to ensure reliability and avoid misinterpretation [108,109,110,111,112].

Despite their predictive potential, AI-based models are often criticized for limited interpretability. Opaque “black-box” outputs may hinder clinical adoption, particularly in high-stakes decision-making. Interpretability is, therefore, a key requirement for implementation in oncology [113,114].

Explainable artificial intelligence (XAI) methods aim to improve transparency by quantifying how individual variables contribute to model predictions. Common approaches include feature importance measures, partial dependence plots, accumulated local effects, and Shapley additive explanations (SHAP), which provide both global and individual-level interpretations [115,116,117,118,119,120].

In this context, biologically interpretable variables such as immune-inflammatory biomarkers may enhance the credibility of model outputs. Markers such as SII are routinely measured and clinically familiar, which facilitates interpretation within multivariable models [121,122].

However, variable importance does not imply causality. XAI methods describe associations within a given dataset but do not establish mechanistic relationships. Interpretation of model outputs, therefore, requires clinical context and independent validation to avoid overinterpretation or bias [113,116,121].

### 4.5. Challenges and Limitations of AI-Based Prognostic Models

Despite ongoing advances, several challenges limit the clinical implementation of AI-based prognostic models. A major concern is overfitting, particularly in datasets with limited sample size, low event rates, or high predictor dimensionality [123,124]. Model performance may, therefore, be overestimated in internal validation and fail to generalize to independent cohorts.

Data quality and heterogeneity represent additional challenges. Variability in data acquisition, feature definitions, and preprocessing can affect model reproducibility and stability across studies [123,124]. Differences in laboratory standards, imaging protocols, and patient populations further limit transportability between institutions [76,125].

External validation remains essential but is often insufficiently performed. Models developed in single-center or highly selected cohorts may not maintain performance in broader clinical settings [17,82].

Implementation in clinical practice also requires appropriate infrastructure, integration with existing workflows, and clinician engagement. Ethical and regulatory considerations, including data privacy, algorithmic bias, and accountability, must be addressed to ensure responsible use [126,127].

AI-based prognostic models should, therefore, be regarded as decision-support tools that complement, rather than replace, clinical judgment by providing probabilistic risk estimates [128,129,130]. This perspective is consistent with broader ethical and regulatory considerations, which emphasize that AI systems in healthcare must support—not substitute–physician responsibility and accountability in decision-making [131].

From a multidisciplinary standpoint, Amann et al. [132] argue that explainability and human oversight are fundamental to ensuring that AI systems are used appropriately in clinical contexts, reinforcing the role of clinicians as final decision-makers [132]. Furthermore, evidence from comparative studies, such as the meta-analysis by Liu et al. [133], shows that while AI models can achieve performance comparable to healthcare professionals in specific diagnostic tasks, their optimal use lies in augmenting clinical expertise rather than replacing it [133].

Together, these findings support the view that AI-driven prognostic tools should be integrated into clinical workflows as supportive instruments, enhancing risk stratification and decision-making while preserving the central role of clinical judgment and responsibility [131,132,133].

## 5. Integrating the Systemic Immune-Inflammation Index into AI-Based Prognostic Models

Integrating the Systemic Immune-Inflammation Index (SII) into AI-based prognostic systems combines a routinely available host-derived biomarker with data-driven analytical methods. SII reflects systemic immune-inflammatory status, while artificial intelligence enables joint analysis of heterogeneous variables relevant to cancer outcomes [134,135]. Owing to its low cost and widespread availability, SII can be incorporated across tumor types and clinical settings, facilitating large-scale data aggregation and external validation [136]. As a clinically familiar laboratory index, it may also support interpretation of individualized risk estimates [137].

In survival analysis, SII is most appropriately modeled as a continuous variable, preserving information lost through dichotomization and enabling flexible estimation of associations [70,71]. When included in multivariable models, potential redundancy should be considered. Simultaneous inclusion of SII and its individual components or related inflammatory indices may introduce multicollinearity and affect model stability [138,139]. Appropriate variable selection and sensitivity analyses are, therefore, required.

Beyond baseline assessment, SII may be evaluated longitudinally using serial measurements to capture temporal variation during treatment or disease progression. Although conceptually attractive, such approaches remain insufficiently validated and require prospective evaluation [140,141]. The prognostic impact of SII may also vary across disease stage, treatment modality, and molecular subtype [142].

**Figure 2:**
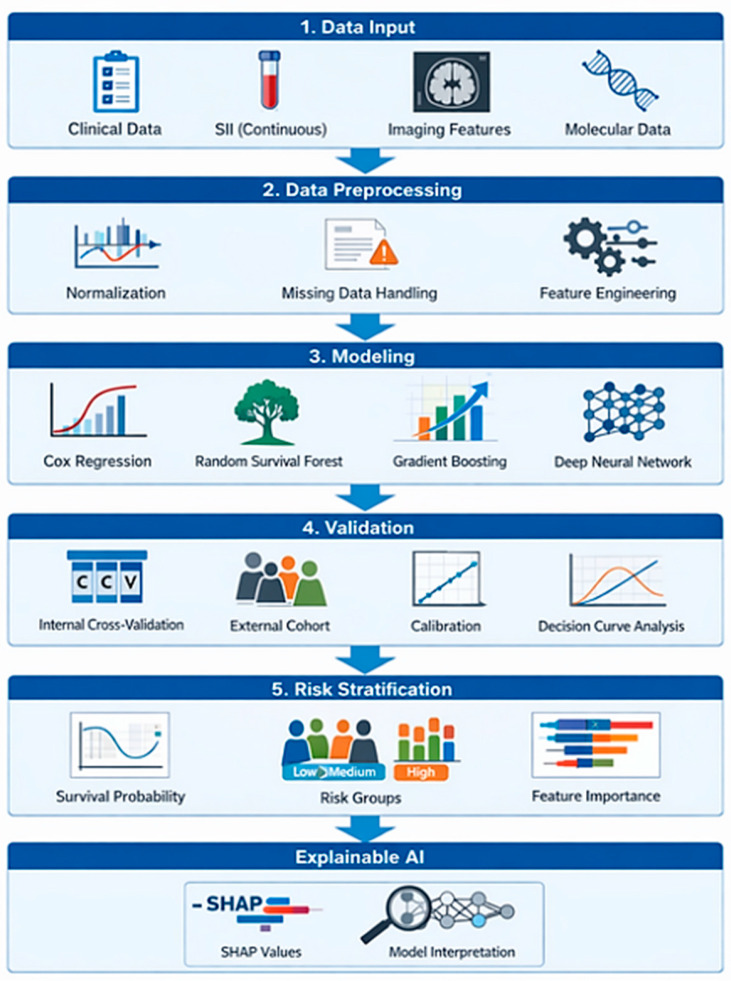
Technical workflow of an AI-based survival modeling pipeline integrating the Systemic Immune-Inflammation Index (SII). This figure was created in BioRender. Mierzejewska, A. (2026) (Available online: https://BioRender.com/v4a9dux; accessed date: 13 March 2026).

A structured modeling pipeline for incorporating SII into prognostic systems is illustrated in Figure 2. This framework includes data harmonization, feature selection, multicollinearity assessment, internal validation, and independent external validation. These steps are essential to ensure robustness and transportability across clinical settings.

Within multivariable models, SII provides information on systemic immune status that is not directly captured by tumor-derived features [143,144,145]. Its contribution should, therefore, be evaluated empirically rather than assumed.

Beyond statistical significance, the incremental predictive value of SII should be formally assessed. Demonstrating independent association does not necessarily imply meaningful improvement in risk prediction. Evaluation should, therefore, include changes in discrimination, calibration, and clinical utility when SII is added to established prognostic models [146,147].

Discrimination can be assessed using concordance indices, although improvements should be interpreted cautiously, particularly in structured datasets where conventional models may perform similarly [14,82]. Calibration is essential to evaluate agreement between predicted and observed outcomes and should be examined before and after inclusion of SII [82,109]. Clinical usefulness should be assessed using decision-analytic approaches such as decision curve analysis, which determine whether model extensions translate into meaningful changes in clinical decision-making [68,71].

As illustrated in Table 2, independent multivariable association does not necessarily correspond to improved predictive performance. Current evidence supports the prognostic relevance of SII; however, robust demonstration of incremental benefit beyond established models remains limited. Explainability methods may identify SII as an influential predictor in specific datasets [146,147,148]. Such findings reflect statistical contribution within a model but do not establish causality [149]. Interpretation, therefore, requires clinical context and biological plausibility.

**Figure 3:**
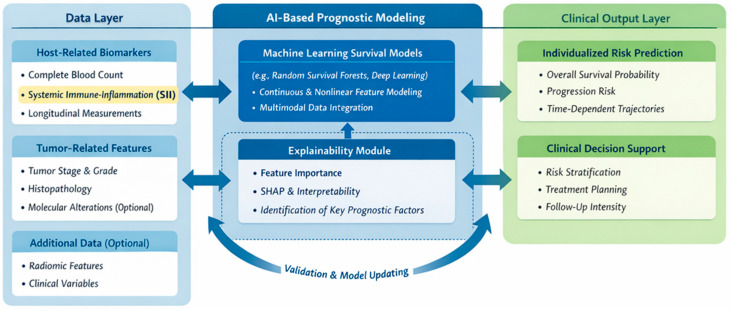
Multilayer conceptual framework for integrating the Systemic Immune-Inflammation Index into AI-based prognostic models in oncology. This figure was created in BioRender. Mierzejewska, A. (2026) (Available online: https://BioRender.com/seupuxr; accessed date: 13 March 2026).

A structured implementation pathway includes model development in representative cohorts, internal validation, independent external validation, and evaluation of both discrimination and calibration prior to clinical use [113,122,150,151]. Ongoing performance monitoring and model updating are required as clinical practices evolve (Figure 3).

Although the layers are based on similar underlying data types, they differ in their functional roles. The Data Layer represents raw clinical and biological inputs generated in routine practice. The AI-based Layer transforms these inputs using algorithmic approaches to produce predictive structures and risk estimates. In contrast, the Clinical Output Layer contextualizes these results within clinical decision-making. While overlap exists due to shared data sources, the layers differ in terms of processing, abstraction, and application.

Clinically, incorporating systemic immune-inflammatory status alongside tumor characteristics may support pre-treatment risk stratification and individualized therapeutic planning [137,138,146]. Longitudinal incorporation of SII may enable dynamic risk assessment during treatment, although prospective validation remains essential [140,141,142]. In research settings, such models may support trial stratification and exploratory analyses of treatment response [143,144,145,149].

SII should, therefore, be considered as one component of a multivariable, externally validated prognostic system rather than as an independent determinant of outcome. When appropriately integrated, it may contribute clinically interpretable information to AI-based risk estimation in oncology.

## 6. Clinical Implications and Translational Potential

The Systemic Immune-Inflammation Index offers practical advantages, including routine availability, low cost, and reproducibility across healthcare settings [11,45,59]. Its translational value is maximized when incorporated into validated multivariable prediction systems rather than applied in isolation [14,44]. Importantly, such integration should be supported by demonstrable incremental predictive performance beyond established clinicopathological models, rather than by statistical association alone.

In pre-treatment assessment, models integrating SII with tumor characteristics may support more refined risk stratification and individualized therapeutic planning [11,14,45]. These approaches may help identify patients who require intensified monitoring or alternative strategies, while also informing de-escalation in selected low-risk contexts [44,67,109]. Importantly, such models should provide probabilistic estimates that support shared decision-making rather than replace clinical judgment [97,134,149].

In longitudinal settings, serial assessment of SII may contribute to dynamic risk evaluation during treatment. Changes in systemic immune-inflammatory status may reflect therapeutic response or disease progression [64,87,141]. Time-updated models could enable earlier identification of patients at increased risk, although prospective validation remains necessary [2,34,103,142].

In clinical research, incorporating SII into multivariable models may improve trial stratification, reduce outcome heterogeneity, and enhance endpoint prediction [44,85,134]. These approaches may also support exploratory analyses of immune-inflammatory correlates of treatment response and inform biomarker-driven study design [24,51,149].

Routine implementation requires standardized data acquisition, appropriate digital infrastructure, and clinician engagement [109,123]. In the context of AI-based prognostic models, ethical and regulatory considerations acquire additional importance. Issues such as algorithmic transparency, potential bias in training data, and accountability for model-informed decisions are particularly relevant when risk estimates may influence clinical management. Ensuring interpretability, external validation, and clear delineation of clinical responsibility is, therefore, essential for safe implementation [102,126,130]. Ongoing evaluation and model updating are required as clinical practice evolves.

From a clinical perspective, integrating simple and routinely available biomarkers such as SII into advanced modeling frameworks represents a pragmatic step toward more applicable precision oncology. Rather than replacing clinical judgment, such models may support more structured and transparent decision-making.

## 7. Limitations and Challenges

Several biological, methodological, and implementation-related limitations should be acknowledged [7,46].

Biologically, SII is a non-specific marker of systemic immune-inflammatory status. Neutrophil, platelet, and lymphocyte counts are influenced by infections, chronic inflammatory conditions, comorbidities, and medications, limiting specificity for cancer-related processes [4,51,53,54].

Beyond non-malignant inflammation, SII may also reflect broader systemic vulnerability rather than tumor-specific biology. Frailty, sarcopenia, cachexia, and age-related immunosenescence are associated with alterations in circulating immune cell profiles and may confound associations between SII and survival outcomes [57]. Elevated SII may, therefore, act as a surrogate of impaired physiological reserve rather than a direct mediator of tumor progression.

Reverse causality represents an additional concern. Advanced disease and occult metastases may induce systemic inflammation, leading to elevated SII values. As a result, SII may partly reflect underlying disease burden rather than independently influencing prognosis.

Treatment-related factors further complicate interpretation. Chemotherapy, corticosteroids, granulocyte colony-stimulating factors, immunotherapy, and radiation can alter peripheral blood counts. Both baseline and on-treatment SII may, therefore, reflect a combination of host biology and therapy-related effects. Failure to account for sampling timing may introduce bias.

These considerations indicate that SII should be interpreted as a composite systemic marker influenced by tumor biology, host vulnerability, and treatment-related factors rather than as a tumor-specific biomarker.

Methodologically, most evidence derives from retrospective studies with heterogeneous populations, tumor types, and analytical strategies. Variability in laboratory standards, sampling timing, and statistical approaches limits comparability. Study-specific dichotomization thresholds further reduce reproducibility and may inflate effect estimates [70,74,83].

AI-based prognostic models introduce additional challenges related to overfitting, dataset quality, and transportability. Performance estimates from internal validation may not generalize to independent cohorts, underscoring the need for rigorous external validation and transparent reporting [17,82,138]. Model outputs also require careful interpretation, as feature importance does not establish causality [113,115,132].

Implementation requires appropriate digital infrastructure, standardized data pipelines, clinician engagement, and clear governance structures. Ethical and regulatory considerations, including data privacy, bias mitigation, and accountability, must be addressed prior to routine deployment [44,83,126].

Finally, the conceptual nature of this review and the absence of quantitative synthesis limit direct comparison of modeling strategies. The proposed framework should, therefore, be considered hypothesis-generating pending prospective validation [70,74].

## 8. Future Directions

Future research should prioritize prospective, multicenter validation with standardized data collection and reporting [68,76,109]. Continuous modeling of SII within multivariable systems may improve reproducibility and transportability across clinical settings [79,90].

Longitudinal incorporation of serial SII measurements represents a promising approach to dynamic risk prediction, although prospective confirmation of clinical benefit remains necessary [134,141].

Integration with emerging biomarker modalities—including circulating tumor DNA, immune profiling, radiomics, and multi-omics platforms—may further refine risk stratification by combining systemic and tumor-derived information [36,145].

Advances in explainable AI and adherence to reporting standards such as TRIPOD-AI and CONSORT-AI will be essential to improve transparency, reproducibility, and comparability of prognostic models [113,130,132,150].

Progress in this field will depend on interdisciplinary collaboration among clinicians, data scientists, and translational researchers to ensure that analytical advances translate into clinically meaningful outcomes.

## 9. Conclusions

The Systemic Immune-Inflammation Index represents a readily available indicator of systemic immune-inflammatory status with reproducible prognostic associations across solid malignancies. However, its clinical utility is maximized when incorporated into rigorously developed and externally validated multivariable prediction models rather than applied as a dichotomized standalone marker.

Embedding biologically interpretable host-derived biomarkers within explainable AI-based survival models enables more context-sensitive risk estimation. Such systems should be regarded as decision-support tools that complement clinical expertise by providing probabilistic risk estimates derived from integrated data sources. Realizing their potential requires prospective validation, standardized reporting, and careful implementation.

Structured integration of immune-inflammatory biomarkers into AI-based survival models may contribute to more precise and clinically relevant risk stratification in oncology. Incorporating both baseline and longitudinal representations of systemic immune-inflammatory status may further enhance their clinical relevance, although prospective validation remains essential.

## Figures and Tables

**Table 1:** Comparison of selected peripheral blood–based immune-inflammatory biomarkers used in oncology.

Biomarker	Components	Biological Interpretation	Common Application Context	Main Limitations
NLR	Neutrophils/ Lymphocytes	Balance between systemic inflammation and adaptive immune competence	Baseline prognostic stratification; treatment response estimation across solid tumors	Ignores platelet involvement; limited reflection of thrombocytic processes
PLR	Platelets/ Lymphocytes	Thrombosis-related tumor promotion versus immune surveillance	Prognostic assessment in advanced disease and perioperative settings	Limited inflammatory context; variability across tumor types
LMR	Lymphocytes/ Monocytes	Immune surveillance vs. macrophage precursors activity	Prognosis in selected hematological and solid malignancies	Less robust and consistent across cancer types
SII	Platelets × Neutrophils/Lymphocytes	Integrated inflammation, thrombosis, and immune suppression	Global host-related prognostic stratification; emerging use in multimodal and AI-based prognostic models	Lack of standardized cut-offs; influenced by non-malignant inflammatory conditions

**Table 2:** Representative primary studies and meta-analyses evaluating the prognostic value of SII in solid tumors.

Author	Study Type	Cancer Type	N	Treatment Context	SII Cut-Off	Outcome	HR (95% CI)	Multivariable Adjustment	Incremental Model Comparison	ΔDiscrimination/Calibration Reported
Hu et al. [45]	Primary cohort	Hepatocellular carcinoma	133	Surgical resection	330	OS	2.21 (1.35–3.61)	Yes	No formal base vs. extended model comparison	Not reported
Aziz et al. [47]		Pancreatic cancer	321		440 *		1.88 (1.20–2.95)		No	
Zhong et al. [11]	Meta-analysis	Multiple solid tumors	22 studies (n ≈ 7657)	Mixed	Study-specific		1.69 (1.42–2.01) **	Adjusted HR pooled	Not applicable	
Yang et al. [59]		Multiple cancers	100 studies (n > 19,000)				1.85 (1.63–2.10) **		Not applicable	
Shui et al. [61]		Pancreatic cancer	10 studies (n = 2365)				1.87 (1.49–2.35) **		No	

* cut-off example; varies across studies, ** pooled hazard ratio. Abbreviations: HR, hazard ratio; N, number of patients.

## Data Availability

No new data were created or analyzed in this study.

## Abbreviations

The following abbreviations are used in this manuscript:

AI	Artificial Intelligence
ML	Machine Learning
XAI	Explainable Artificial Intelligence
SII	Systemic Immune-Inflammation Index
NLR	Neutrophil-to-Lymphocyte Ratio
PLR	Platelet-to-Lymphocyte Ratio
LMR	Lymphocyte-to-Monocyte Ratio
CRP	C-Reactive Protein
Mgps	Modified Glasgow Prognostic Score
PNI	Prognostic Nutritional Index
TNM	Tumor–Node–Metastasis staging system
CtDNA	Circulating Tumor DNA
PRISMA	Preferred Reporting Items for Systematic Reviews and Meta-Analyses
SHAP	Shapley Additive Explanations
NSCLC	Non-Small Cell Lung Cancer
PD-1	Programmed Cell Death Protein 1
XELOX	Capecitabine + Oxaliplatin chemotherapy regimen
SEER	Surveillance, Epidemiology, and End Results database
TRIPOD-AI	Transparent Reporting of a Multivariable Prediction Model for Individual Prognosis or Diagnosis–Artificial Intelligence extension
PROBAST	Prediction Model Risk of Bias Assessment Tool
CONSORT-AI	Consolidated Standards of Reporting Trials–Artificial Intelligence extension
SPIRIT-AI	Standard Protocol Items: Recommendations for Interventional Trials–Artificial Intelligence extension
